# Factors Influencing the Estimates of Correlation between Motor Unit Activities in Humans

**DOI:** 10.1371/journal.pone.0044894

**Published:** 2012-09-25

**Authors:** Francesco Negro, Dario Farina

**Affiliations:** 1 Department of Neurorehabilitation Engineering, Bernstein Focus Neurotechnology Göttingen, Bernstein Center for Computational Neuroscience, Georg-August University of Göttingen, Göttingen, Germany; 2 Center for Sensory-Motor Interaction (SMI), Department of Health Science and Technology, Aalborg University, Aalborg, Denmark; University of Alberta, Canada

## Abstract

**Background:**

Alpha motoneurons receive common synaptic inputs from spinal and supraspinal pathways. As a result, a certain degree of correlation can be observed between motoneuron spike trains during voluntary contractions. This has been studied by using correlation measures in the time and frequency domains. These measures are interpreted as reflecting different types of connectivity in the spinal networks, although the relation between the degree of correlation of the output motoneuron spike trains and of their synaptic inputs is unclear.

**Methodology/Principal Findings:**

In this study, we analyze theoretically this relation and we complete this analysis by simulations and experimental data on the abductor digiti minimi muscle. The results demonstrate that correlation measures between motoneuron output spike trains are inherently influenced by the discharge rate and that this influence cannot be compensated by normalization. Because of the influence of discharge rate, frequency domain measures of correlation (coherence) do not identify the full frequency content of the common input signal when computed from pairs of motoneurons. Rather, an increase in sampling rate is needed by using cumulative spike trains of several motoneurons. Moreover, the application of averaging filters to the spike trains influences the magnitude of the estimated correlation levels calculated in the time, but not in the frequency domain (coherence).

**Conclusions:**

It is concluded that the analysis of coherence in different frequency bands between cumulative spike trains of a sufficient number of motoneurons provides information on the spectrum of the common synaptic input. Nonetheless, the absolute values of coherent peaks cannot be compared across conditions with different cumulative discharge rates.

## Introduction

During sustained contractions, motoneurons receive both common and independent synaptic inputs from presynaptic neurons and supraspinal centers [1], [2], [3]. The common inputs alter the membrane potentials of different motoneurons in a correlated way and this slightly increases the probability that pairs of motoneurons discharge action potentials almost concurrently [4], [5], [6]. The motoneurons *integrate* the common synaptic inputs *and discharge* more synchronously than in the absence of common input, since their membrane potentials share common fluctuations. The resulting output spike trains correspond to the results of several sampling processes whose pulse densities follow the amplitude fluctuations of the shared input. To quantify the amount of common input to the motoneuron pool, several correlation measures in the time and frequency domains are used [7], [8], [9], [10]. The approach is based on the measure of the degree of correlation between spike trains discharged by motoneurons, which can be assessed in vivo with motor unit recordings. Because of the highly reliable synaptic connection between motoneuron axons and muscle fibers, in the following we will refer to motoneuron or motor unit spike trains indifferently.

The main assumption behind methods for correlation analysis of output spike trains is that the correlation between output spike trains is proportional to the relative degree of common input that the motoneurons receive (see [11] for review). Therefore, the estimation of the output correlation of motoneuron spike trains may give information about the connectivity between the motoneuron pool and spinal or cortical networks.

Different ways for quantifying the strength of association between spike trains of motoneurons are usually interpreted as indicators of separate underlying physiological mechanisms. For example, the degree of short-term synchronization [9] and the common drive [7] between motor units are both computed from the cross-correlation function of motoneuron spike trains but with different pre-filtering and are usually associated to different types of connectivity between inputs to motoneurons [12].

The correlation analysis between spike trains of neurons has been extensively investigated from the 60s [13], [14]. However, only recently have there been studies that focused on the limitations of these measures for the analysis of connectivity in cortical networks [15], [16], [17], [18], [19], [20], [21], [22]. In this study, we use some of these new findings and adapt them for the interpretation of the correlation and synchronization indexes between alpha motoneuron spike trains.

The correlation between spike trains can be performed in the time (cross-correlation) or frequency (coherence) domain. These analyses have different mathematical properties and are thus influenced differently by the properties of the spike trains. Despite the extensive use of these analysis methods in the study of motor unit physiology (e.g., [8], [23], [24], [25], [26], [27], [28], [29], [30], [31], [32], [33], [34]), there are several issues in the interpretation of measures of correlation between neuronal spike trains that should be considered when discussing motoneuron output correlations. The output correlation depends on the input correlation in a non-linear way because it is also a function of the mean and variance of the current injected in the neuron [16]. This problem practically results in an intrinsic dependency of the correlation measures on the discharge rates of motoneurons [35] and has been addressed by various normalization methods [32], [36] that may remove such dependency in some conditions [35]. However, the response of a neuron to a driven current is essentially a sampling process [37] that submits to the sampling theorem [38] therefore the estimated output correlation inherently depends on the discharge rate, up to a saturation point that corresponds to the number of samples necessary for correctly reconstructing the oscillations of the input current. Since in normal contractions, the common synaptic inputs have a bandwidth above the sampling possibilities of the single motoneuron (∼10–20 pps), the full reconstruction is usually not possible for individual motoneurons. Thus, removal of the dependency of a correlation measure on discharge rate does not necessarily correspond to more accurate estimates of the input correlation. Because the dependency of correlation output on discharge rate and other properties of correlation functions between spike trains have not been investigated theoretically for motor neurons, some physiological mechanisms of motor unit synchronization and common drive are still debated. For example, the dependence of correlated inputs to motoneurons on the force level is based on experimental observations of dependence of synchronization indexes on force when also the motor unit discharge rate varies [39].

Currently, the strength of common input to motoneurons is estimated by many indexes, with various normalizations, in the time or frequency domain, usually using pairs of motor units. The appropriateness of each estimation method and the relations between these methods are not fully clear. Therefore, in this study, we provide an analytical description of the two fundamental aspects necessary for correctly interpreting correlation measures between motor unit spike trains: sampling and filtering. Using theoretical, modeling and experimental analyses, we show that the dependence on discharge rate of the various correlation measures previously proposed is an intrinsic limitation of the sampling process generated by the spiking nature of the motoneurons. Moreover, the dependency on discharge rate introduces a large variability in the estimates that can limit their use in practical applications. Under the assumption of a synaptic noise commonly spread across the motoneuron pool, the equivalent sampling rate can however be increased by the concurrent analysis of populations of motoneurons. Additionally, we describe how different indexes for expressing correlation between output spike trains (e.g., synchronization and common drive indexes) can be interpreted by the application of temporal averaging filters of different lengths to the motoneuron spike trains. Therefore, these indexes represent the same phenomenon in different frequency bandwidths, so that the same information can be obtained by a frequency analysis approach rather than by several time-domain methods.

## Materials and Methods

### Theory

The effect of discharge rate (sampling) on the level of correlation estimated at the cell output has been recently studied for cortical neurons [16] and can be described by a mathematical derivation under the assumption of leaky integrate-and-fire neurons and Gaussian noise. This derivation is partly valid also for motoneurons, since they can be approximated to some extent by this model (Goroso, Cisi, Kohn 2000).

The input currents delivered to a population of motoneurons can be described as:
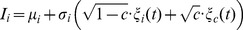
(1)where *c*, *µ_i_*, *σ_i_*, *ξ_i_(t)* and *ξ_c_(t)* are, respectively, the input correlation, the mean and standard deviation of the synaptic input current and the realizations of independent and common Gaussian noises for each motor neuron *i*. With this formalism, the input correlation is defined as the proportion of variance of a common synaptic input that is shared by different motor neurons. The expression for the output correlation between spike trains of two motoneurons receiving the inputs described by Eq. (1) is [16]:
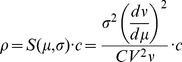
(2)where ρ is the output correlation and μ, σ, ν, CV are, respectively, the mean value of the synaptic input current, its standard deviation, the average discharge rate of the motoneuron and the coefficient of variation for the interspike-interval (ISI). Eq. (2) shows that the output correlation is related to the input correlation by the scaling factor S(μ,σ), which for cortical neurons has been referred to as correlation susceptibility [16]. This function depends on the statistics of the input current and the discharge rate of the neuron. Since the statistics of the input current cannot be reliable estimated in vivo, the relation between ρ (measured correlation) and c (actual input correlation) is essentially unknown. Moreover, the dependency on discharge rate of the estimated output correlation cannot be easily eliminated due to the relation between input current and discharge rate. Eq. (2) is valid only in the “low-correlation” regime (c<0.3), a level comparable with the correlation that can be measured in vivo in motoneurons, whereas it can be shown that for high correlations the dependence on the discharge rate tends to vanish [20]. Eq. (2) indicates that the dependence of the output correlation on the discharge rate is a property of the spiking nature of the neurons and not a bias due to the increase probability of coincident spikes with increasing discharge rates. In practice, indexes extracted from the motoneuron spike trains can only estimate the output correlation ρ. The aim of this estimate is to get an accurate representation of the input correlation c. Eq. (2) indicates the association between the variable that can be measured (ρ) and the intrinsic correlation level (c), which is the unknown physiological strength of common input. While c is fixed in a given condition, ρ depends on other factors, in addition to c, and thus can vary even if c does not.

The above considerations are valid for cortical as well as spinal neurons and can be interpreted also considering the sampling process associated to the motoneuron discharging. Higher discharge rates imply a better sampling of the input current [37], [40], [41] and therefore a better reconstruction of the input signals, that results in greater values of the estimated output correlation *ρ*. However, the bandwidth of the input current to the motoneuron pool is unknown and thus it is not possible to estimate the minimal sampling rate (discharge rate) that would allow a measure without under-sampling. For this reason, the estimated output correlation *ρ* may provide limited information on the actual amount of correlation in input *c*, as it will be shown in the Results by both simulations and experimental tests.

Under the assumption of a common noise shared across a population of motoneurons, the pooling of several motoneuron spike trains in a composite spike train (CST) can in principle improve the estimation of correlation because it increases the average sampling rate of the common input to motoneurons [37], [41]. However, the measure of correlation estimated from pooled spike trains increases monotonically with the number of spike trains until saturation [18]. This effect is easily explained by observing that the summation process for generating the CST performs an averaging that removes the uncorrelated part of the output signals so that what remains is entirely correlated. For these reasons, the estimation of the correlation coefficients calculated from CST will overestimate the actual correlation value and will depend on the number of spike trains used for the estimation.

For the estimation of the output correlation *ρ*, time and frequency domain measures can be used. If we indicate as *x_i_(t)* the output spike train of the i-th motoneuron, the covariance function between two spike trains is defined as [22]:
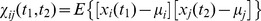
(3)


If instead of considering a raw spike train, we generate a filtered version of it *y_i_(t)*, defined as:
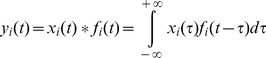
(4)where *f_i_(t)* is the impulse response applied to the *i*-th spike train, the covariance function becomes:
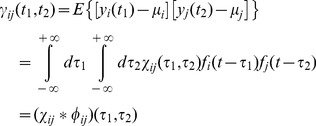
(5)where φij is the deterministic auto-correlation function of the filter impulse response. If the signals yi(t) and yj(t) have finite energy, a normalized measure of correlation can be calculated as:
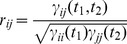
(6)that is limited between [−1,1]. Note that according to these notations, the common drive indexes [7] and the synchronization indexes [9], [36] are defined in the same way but with a different filtering of the spike trains. These time-domain measures depend on the transfer function of the filter applied, therefore the estimated output correlation can differ depending on the length of the filter applied.

In the frequency domain, the coherence function is defined as the Fourier transform of the cross-correlation, which after normalization can also be written as:
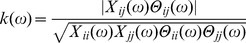
(7)where *X_ij_(ω)* is the cross-spectrum (the Fourier transform of the cross-correlation function) of the two spike trains, *Θ_ij_(ω)* the cross-spectrum of the filter kernels (the filters applied to the spike trains) and *X_ii_(ω)*, *X_jj_(ω)*, *Θ_ii_(ω)*, *Θ_jj_(ω)* the respective autospectra.

Contrary to the cross-correlation function, the coherence function is independent of the filter transfer functions, as is shown in the following:
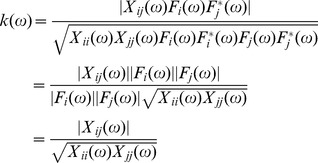
(8)


Therefore, in the frequency range defined by the applied filter, the coherence measure is independent of the filter used, contrary to time-domain measures. Filtering applied to spike trains provides different time-domain indexes of correlation but does not change the coherence values in the filter bandwidth. Practically, filtering defines the subband of analysis in the coherence function so that filtering the spike trains is equivalent to limit the coherence function to a subband defined by the bandwidth of the filter, without changing the value of the coherence function in such subband.

From the above theoretical considerations, it is concluded that 1) the scale factor between the measured output correlation between spike trains and the input correlation cannot be estimated and depends on the discharge rate (Eq. (1)); 2) filtering spike trains impact the time-domain measures of output correlation (Eq. (5)) but not the frequency measures (Eq. (8)), so that filtering spike trains is equivalent to a subband analysis of the correlation in the frequency domain. The impact of these theoretical conclusions in estimating the strength of correlation between motor unit spike trains was tested by simulations and experimental analyses.

### Simulations

Correlation Indexes were Computed from Signals Simulated with a Realistic Motoneuron Model.

#### Motoneuron model

The motoneuron model was a modification of that described by [42]. It consists of two compartments and six conductances. The pulse-based simplification used in the original model was removed in the present study and a full formulation, previously proposed in another model [43], was used instead. The motoneuron parameters were the same as used by [42] (their Table 2) and selected according to an exponential distribution over the pool of motoneurons [44]. This model was chosen because it provides motor unit behaviours similar to those observed experimentally. The number of motoneurons was chosen equal to 300, which is similar to histological findings in the abductor digiti minimi muscle [45], which was the muscle used for the experimental analyses (see below).

**Figure 1:**
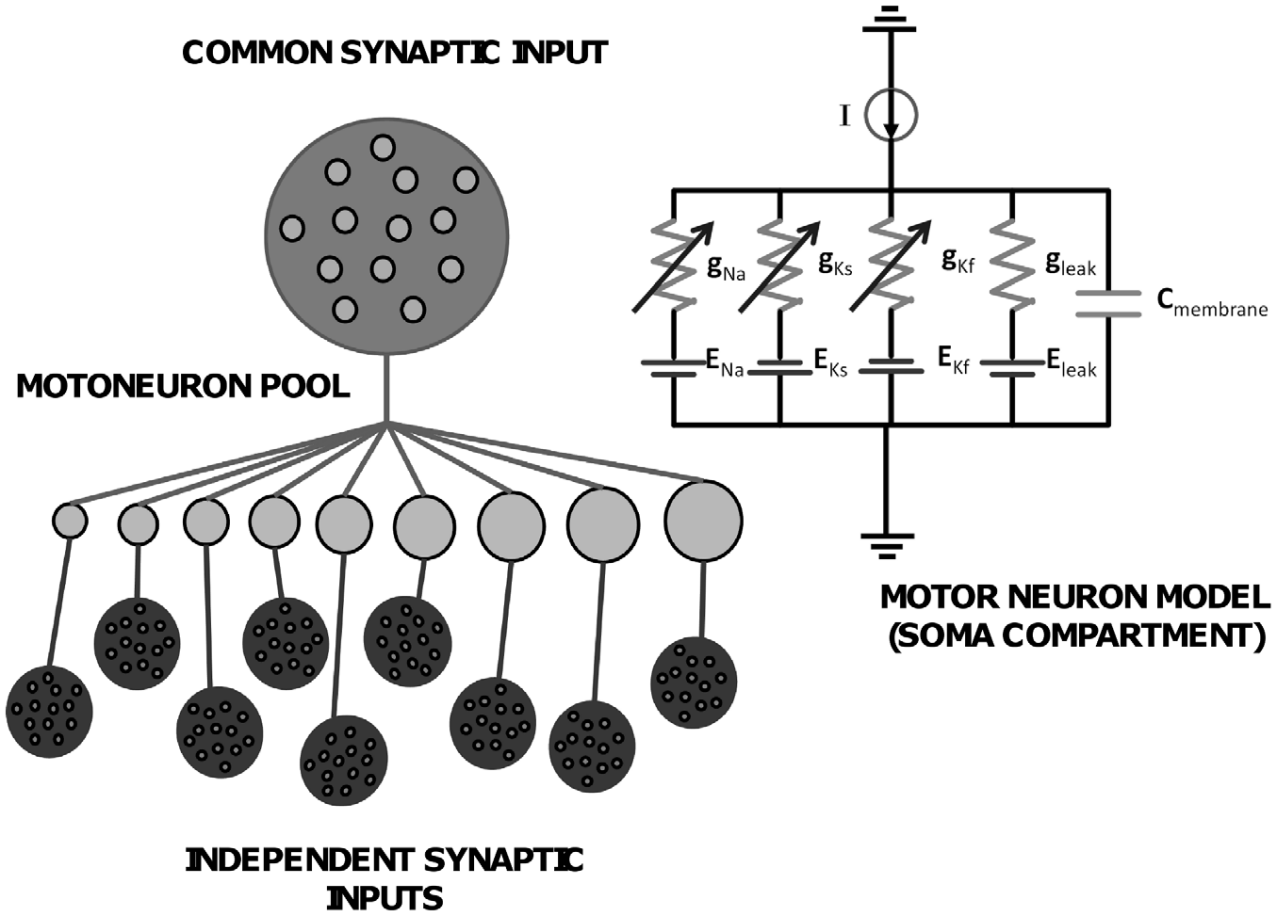
Schematic representation of the model. *A*, types of synaptic inputs incorporated in the model. *B*, equivalent circuit of the motoneuron model (soma compartment).

The input to the motoneuron pool was divided into two components: one common component resembling a shared synaptic input to the entire motoneuron pool and an independent component for each motoneuron. The *common input* (CI) and *the independent input (IN)* were modelled as a band-limited (0–100 Hz) white Gaussian noise [46]. This large bandwidth was chosen to be as general as possible, even if the experimental recordings usually show some specific harmonics. Anyway, the main conclusions of the manuscript are independent of the selected bandwidth. The common and independent inputs were weighted in the same way for small and large motoneurons and the current was injected uniformly across the motoneuron pool. The input to the motoneuron pool was the linear combination of the two input sources. The total variance of the stochastic input was a percentage of the steady-state drive in order to obtain a coefficient of variation for the interspike interval (ISI) of approximately 15% [46]. A schematic representation of the motoneuron model is shown in Figure 1.

The stochastic input current was applied to the soma compartment as an injected current. All motoneurons received the same amount of synaptic input. Therefore, the motoneuron model did not have limitations in discharge rate and did not describe tonic firing in the absence of synaptic input. These simplifications are acceptable in the current study since discharge rate saturation has a limited effect at low level of mean input current (as in the simulations presented in the Results). Our model did not incorporate voltage-dependent dendritic channels and purely inhibitory inputs because an exhaustive study on these inputs and correlation output is available [47].

The full model was implemented in Matlab. The system of differential equations for the motoneuron model was solved with the Adams-Bashforth-Moulton PECE solver [48], using optimized time steps within intervals of 1 ms. Each simulation was 100-s long.

### Experimental Analysis

Motor unit spike trains were experimentally analysed in healthy subjects.

#### Subjects

Eight healthy men participated in the experiments (mean ± SD, age: 25.7±2.3 yrs; range, 23–31 yrs). The experiments were conducted in accordance with the Declaration of Helsinki and approved by the ethics committee of Nordjyllands (approval number N-20090019). All participants self-reported to be right handed and signed a written informed consent form before inclusion.

#### Recordings

Single motor unit action potentials were recorded from the abductor digiti minimi muscle with Teflon-coated stainless steel wires (diameter 0.1 mm; A-M Systems, Carlsborg, WA) inserted into the muscle with 25-gauge hypodermic needles. To increase the sampling size of the motor unit population, three pairs of wires were placed approximately 5 mm apart in the transverse direction in the proximal portion of the muscle. The needles were inserted and removed after the insertion, leaving the wires inside the muscle. Each wire was cut to expose the cross section of the tip without insulation. The three bipolar intramuscular EMG signals were amplified (Counterpoint EMG, Dantec Medical, Skovlunde, Denmark), band-pass filtered (500 Hz to 5 kHz), and sampled at 10 kHz. The position of the wires was slightly adjusted before starting the recordings and when the signal quality was poor, which occurred rarely, a new pair of wires was inserted.

In order to increase the sample size of the motor unit population even further, in only one subject (Subject 7), two additional needle insertions (for a total of 5) were performed. This could not be repeated in all subjects because of discomfort. Recordings from this subject were used to prove experimentally the saturation properties of measures of correlation output.

#### Procedures

The subject was seated on an adjustable chair with the right arm extended in a force brace (Aalborg University). The fifth finger was fixed in the isometric device for the measurement of finger-abduction forces. The forearm and the four digits were secured with Velcro straps. The force produced by the fifth finger was measured using two force transducers (Interface, Arizona USA), one in the transverse plane (abduction force) and the other in the sagittal plane (flexion force). The force signal was sampled at 10 kHz. Visual feedback on the finger abduction force was provided on an oscilloscope.

The subjects performed three maximal voluntary contractions (MVCs) of finger abduction with a rest of 2 min in between MVCs’. The maximum force achieved during the maximal contractions was considered as the reference MVC. The electrodes for EMG recording were then mounted, as described above. The subject increased the force level to a value for which the degree of interference in the signal was sufficient to identify 3–5 motor units per wire and low enough to assure accurate signal decomposition and to limit fatigue. At this level the subject performed a sustained contraction lasting 100 s.

The subject had visual feedback on force during the contractions and was asked to maintain the target force level as stable as possible. During each contraction, the flexion force was monitored and contractions with flexion force higher than one percent of the force generated during the MVC attempt were repeated.

#### Decomposition of experimental signals

Individual motor units were identified from the intramuscular EMG signals recorded from the three (five for subject #7) locations in the muscle by the use of a decomposition algorithm [49]. This interactive algorithm (EMGLAB) includes a user interface for manually editing and verifying the correctness of the spike trains. Each motor unit spike train was manually edited using this visual interface by an experienced operator and any unusually long (>250 ms) or short (<20 ms) inter-spike intervals (ISIs) were manually inspected for checking potential discrimination errors. From the results of the decomposition, spike trains of individual motor units were obtained with a sampling rate of 1000 Hz. The CST (composite spike train) was defined as the sum of individual spike trains, as for the simulated signals.

#### Synchronization and coherence analysis in simulated and experimental signals

The degree of spike train correlation was calculated using the cross-correlation function on the simulated and experimental motor unit spike trains. The calculation was computed on the raw spike trains (equivalent to a rectangular filter of 1 ms duration, given a sampling frequency of 1000 Hz) and on the spike trains filtered using 150 ms and 400 ms Hann windows. From the raw and filtered spike trains, several indexes of correlation were extracted.

The cross-correlation function for the raw spike trains (bin width: 1 ms) were calculated between −100 ms prior and 100 ms after the discharge of the reference unit [6], [28], [36]. This particular interval was chosen for comparison with the cited studies since it is the one commonly used in those. The cumulative sum (CUSUM) technique was used to assess the location of the peak of the histograms [50]. The time bin where the cumulative sum exceeded 3 SDs with respect to the mean calculated over the first 50 bins was set as the location of a significant peak [23]. Histograms with a mean count less than 4 were not analyzed [12]. The common input strength (CIS) index [36] was computed as the number of synchronous discharges of the motor unit pair in excess of chance, divided by the duration of the analyzed interval. The Synchronous Impulse Probability (SIP) index was also derived from the analysis of raw spike trains. This index is defined as the number of counts in the peak above the chance divided by the number of spikes used to compute the cross-correlation function [9]. The CIS and SIP are commonly used to quantify the degree of short-term synchronization in motor unit spike trains [51].

Indexes of correlation were also obtained by filtering the spike trains. From the spike trains filtered with 400 ms Hann windows, the common drive index (CDI) was calculated after the application of a detrend high-pass filter with cut-off frequency 0.75 Hz (zero-phase filter),
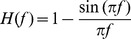
(9)to remove offsets and trends, as proposed in previous studies [7], [52]. The strength of common drive was computed on these filtered spike trains over intervals of 5-s and averaged over the full signal duration. To compare the effect of different low-pass filters, a Hann window of 150 ms duration was also used in the analysis. Only peak values of the cross-correlation function corresponding to time delays in the interval ±100 ms were considered for this analysis [53].

The coherence was also calculated between pairs of (filtered) motor unit spike trains as in Eq. (7).For computing the coherence in simulated and experimental signals, the recording segments were divided in intervals of 5-s and 1-s duration from which the power spectra were estimated with the periodogram (Hanning window). The confidence limit for coherence values was [54]:
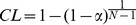
(10)where N is the number of segments used in the calculation and α is the level of confidence.

#### Statistical analysis of experimental data

For the experimental data, linear regression analysis was used to assess the relation between CIS, SIP, or CDI and the geometric mean of discharge rate values. Data are reported as mean and SD. Significance was accepted for *P* values smaller than 0.05. The significance level for coherence functions was computed according to Eq. (10) with *α* = 0.05.

## Results

The results will indicate the effect of sampling and filtering when computing output spike train correlations. For this purpose, results from the experiments and simulations are presented together and interpreted based on the theoretical framework described above.

From the experiments, a total of 94 motor units (average: 11±1, with a maximum of 16 motor units detected from subject #7 from 5 recording sites) were recorded from the eight subjects. 477 pairs (average: 59±13, min-max: 43–74) of motor units showed a significant level of synchronization, according to the criteria described in Methods. The average discharge rate of the motor units analyzed was 12.5±2.1 pps, with a range between 7.1 and 19.5 pps. The average CoV for ISI was 21.0±2.5%. The average level of force produced by the subjects was 4.5±0.5% MVC. Table 1 shows the results for the individual subjects.

**Table 1:** Motor unit statistics for the 8 subjects.

	Force (% MVC)	N. Motor Units	DR (pps)
**Sub. 1**	4.5	11	9.29±3.5
**Sub. 2**	4.3	11	15.1±2.53
**Sub. 3**	5.1	13	14.1±2.21
**Sub. 4**	3.8	10	12.1±1.66
**Sub. 5**	4.1	11	9.6±2.28
**Sub. 6**	4.2	12	11.8±1.76
**Sub. 7**	4.5	16	10.1±2.34
**Sub. 8**	5.1	10	17.6±3.41

MVC: maximal voluntary contraction. DR: average discharge rate in pulses per second.

### The Sampling Issue: Dependence of Correlation Indexes on Discharge Rate

**Figure 2:**
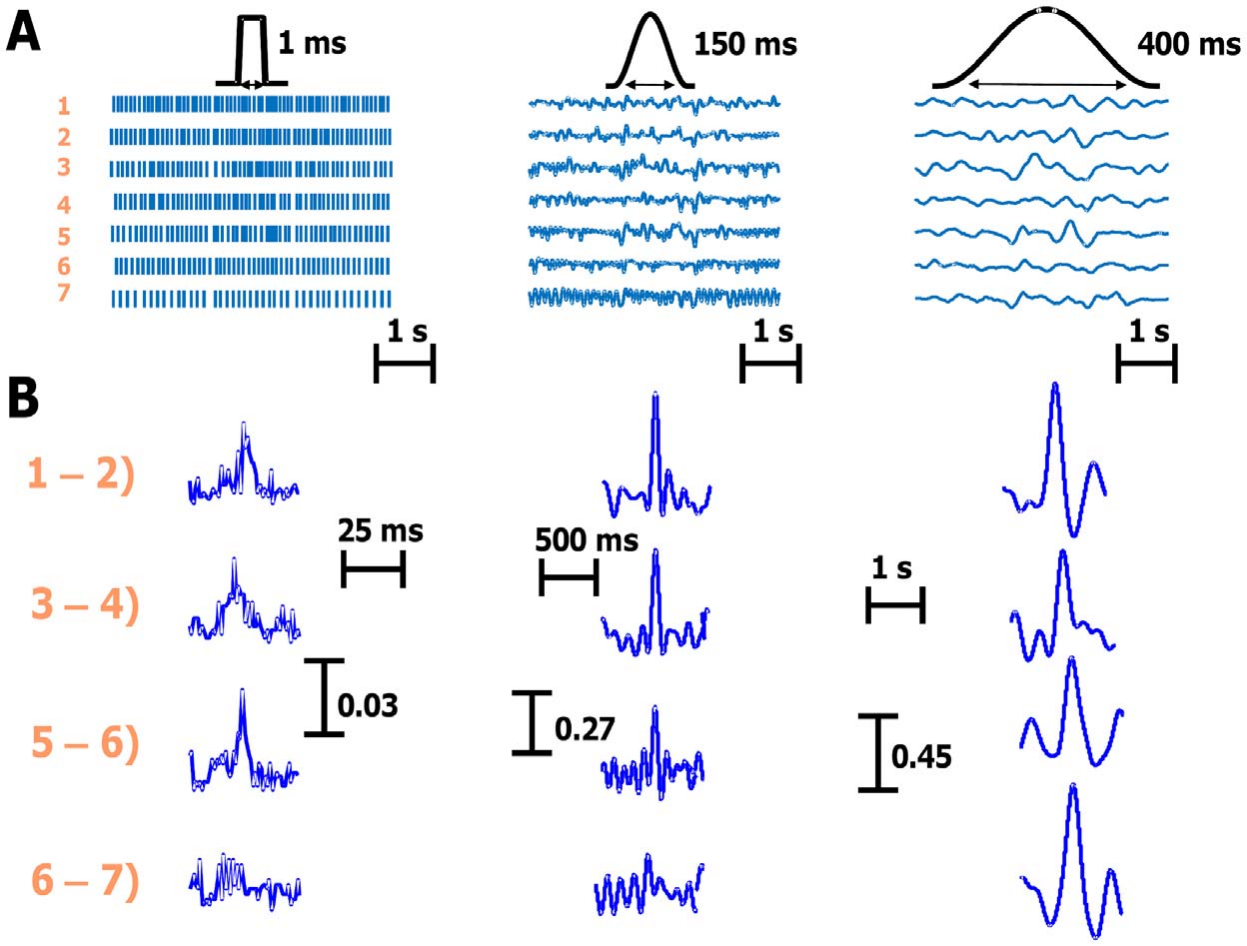
Representative example of the behavior of the correlation measures for seven motor unit spike trains recorded from subject #4. *A*, spike trains of seven motor units filtered with 1 ms, 150 ms and 400 ms duration low-pass windows (shown on top). The spike trains are ordered in decreasing discharge rates (6–13 pps). *B*, cross-correlation functions between pairs of the same filtered spike trains. Note the dependence of the peak correlation on the average discharge rates of the pairs and the length of the filters.

As indicated in Eq. (2), discharge rate inherently influences the output correlation. This was observed experimentally for different filters applied to the spike trains. Figure 2 shows a representative example of seven spike trains recorded from subject 4. Fig. 2A shows the filtered spike trains with the corresponding impulse response of the filters used. The spike trains are ordered from the higher to the lower discharge rates. Fig. 2B shows the cross-correlation function between the filtered spike trains (time-domain analysis). The magnitude of the central peak depended on the pair of motor units chosen for the analysis and thus on the discharge rate of the pair. Variability in the individual results is present (see for example the higher peak of the cross correlation between the 5th and 6th spike trains for the raw data), as expected, but a clear pattern of dependence between correlation peak and discharge rate is evident when the pooled data are analysed, as described below. Moreover, the filter length has also an influence on the estimation since for long filters the dependence on the discharge rate tends to vanish. For example, there is no clear trend for the longest filter in Fig. 2.

**Figure 3:**
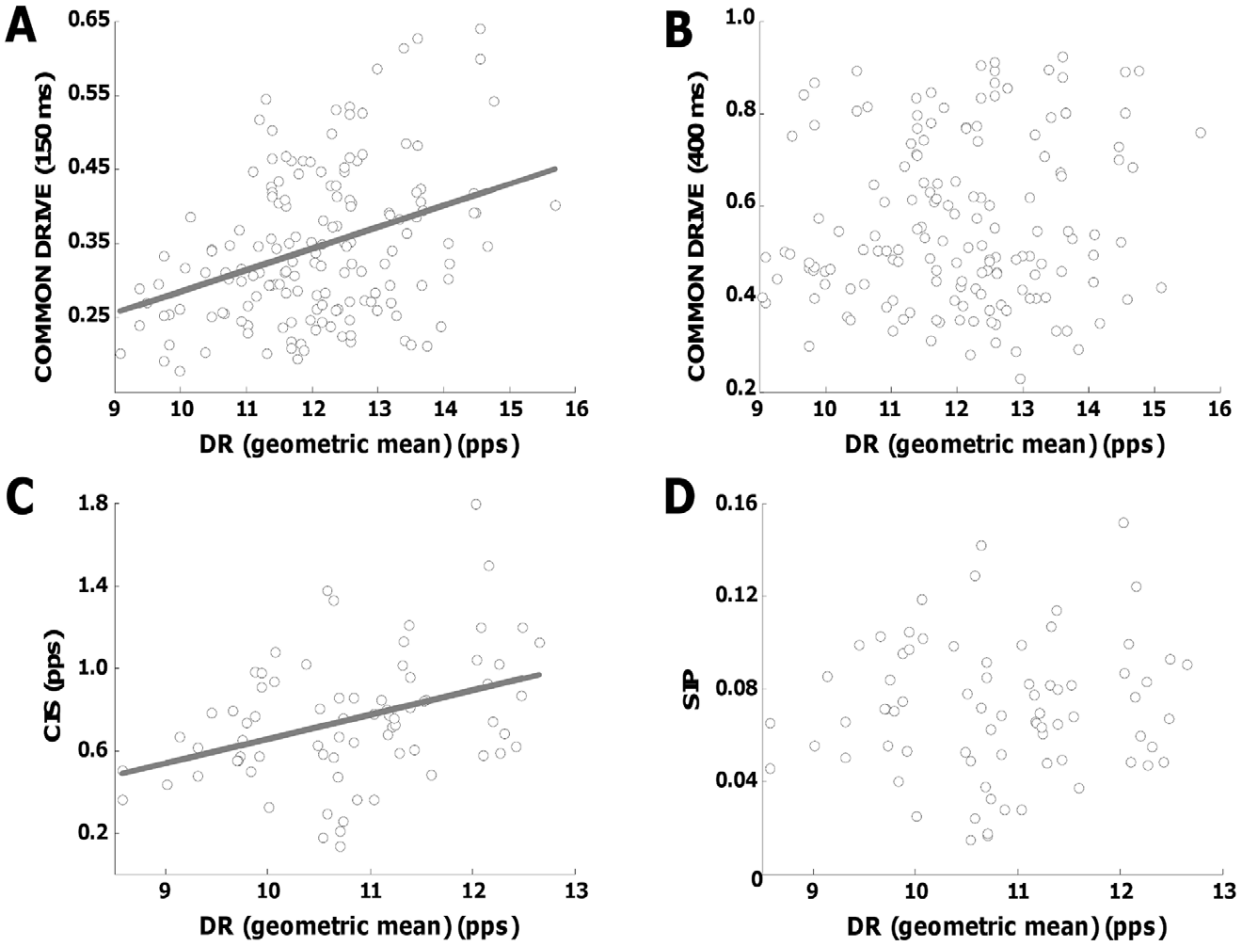
Association between the discharge rates of the pairs of spike trains for four correlation indexes calculated from subject #6. A, scattered plot of the common drive index calculated using an Hann window of 150 ms duration and the geometric mean of the discharge rates of the pairs of spike trains used in the calculation (Rˆ2 = 0.14, P<0.05). B, the same but using a longer window of 400 ms duration (P>0.05). C, scattered plot of the CIS index and the geometric mean of the discharge rates (Rˆ2 = 0.13, P<0.05). D, scattered plot of the SIP index and the geometric mean of the discharge rates (P>0.05).

Figure 3 shows the regression analysis between average discharge rate and correlation indexes performed on a representative subject (subject #6). Figure 3A shows a significant linear relation between the CDI calculated using a short Hann window of 150 ms and the geometric mean of the discharge rates (R2 = 0.13, P<0.001). However, this association was not significant (R2 = 0.02, P  = 0.12) when a longer filter was applied (Hann window of 400 ms) for the same index (CDI), demonstrating a dependence of the behaviour of this correlation index on the filter applied (Figure 3B). The CIS was linearly associated (R2 = 0.14, P<0.001) with the geometric mean of the discharge rates (Fig. 3C) whereas SIP did not show dependence (R2 = 0.006, P  = 0.5) on discharge rate (Fig. 3D). The regression analysis performed on all subjects (Table 2) confirmed these representative results. The same calculations applied to the simulation data showed similar results.

**Table 2:** Correlation coefficients between motoneuron spike trains for the 8 subjects.

	CIS	SIP	CDI (150 ms)	CDI (400 ms)
**Sub. 1**	R^2^ = 0.09, P<0.05	R^2^ = 0.005, P = 0.63	R^2^ = 0.001, P = 0.14	R^2^ = 0.0022, P = 0.61
**Sub. 2**	R^2^ = 0.05, P<0.05	R^2^ = 0.01, P = 0.16	R^2^ = 0.0001, P = 0.85	R^2^ = 0.004, P = 0.07
**Sub. 3**	R^2^ = 0.19, P<0.001	R^2^ = 0.05, P = 0.05	R^2^ = 0.04, P<0.05	R^2^ = 0.005, P = 0.37
**Sub. 4**	R^2^ = 0.04, P<0.05	R^2^ = 0.07, P<0.05	R^2^ = 0.02, P<0.05	R^2^ = 0.00007, P = 0.86
**Sub. 5**	R^2^ = 0.03, P = 0.051	R^2^ = 0.02, P = 0.06	R^2^ = 0.02, P<0.05	R^2^ = 0.0002, P = 0.76
**Sub. 6**	R^2^ = 0.14, P<0.001	R^2^ = 0.006, P = 0.5	R^2^ = 0.13, P<0.001	R^2^ = 0.015, P = 0.12
**Sub. 7**	R^2^ = 0.16, P<0.05	R^2^ = 0.09, P<0.05	R^2^ = 0.046, P<0.001	R^2^ = 0.003, P = 0.35
**Sub. 8**	R^2^ = 0.19, P<0.05	R^2^ = 0.08, P = 0.06	R^2^ = 0.11, P<0.001	R^2^ = 0.03, P = 0.05

CIS: common input strength (pps). SIP: synchronous input probability; CDI (150 ms): common drive index calculated using a hann window of 150 ms duration. CDI (400 ms): common drive index using a window of 400 ms duration.

It is important to notice that all these measures, which are calculated using pairs of motor unit spike trains, suffer from the effect of not complete sampling and therefore show high degree of variability.

These results are explained by different sensitivity of these indexes to filtering and sampling and to their normalization. It is however to be noted that it is not possible to decide which of these indexes is closer to the actual intrinsic correlation degree (*c* in the theoretical derivation), due to the unknown scaling between the estimate of *ρ* (the computed indexes) and *c* (see theoretical derivation).

**Figure 4:**
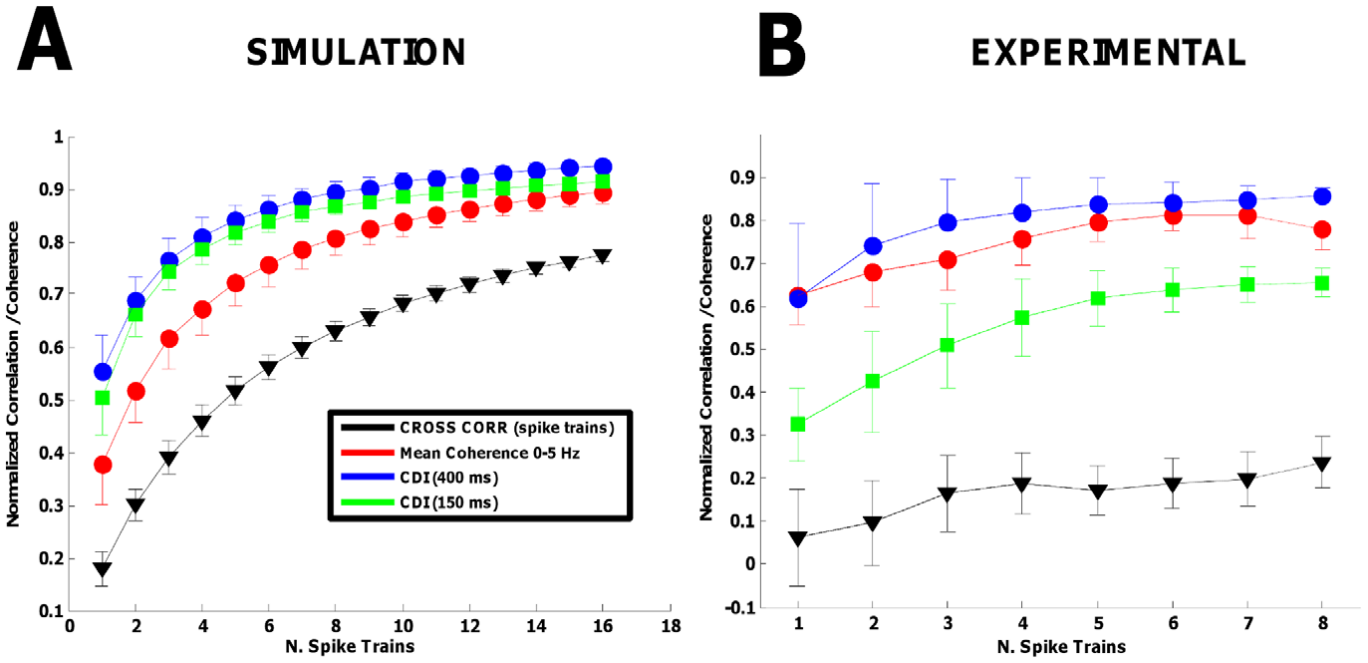
Normalized correlation values and coherence using the pooling of multiple spike trains. *A*, results from the simulations using the band-pass Gaussian noise in the frequency band 0–100 Hz. *B*, results from the experimental recording on Subject n. 7, the one whit the highest number of motor unit spike trains correctly identified. Note a trend of saturation for all indexes when more than 4–5 motoneuronspike trains were used in the calculations.

The dependence on the average discharge rate is a consequence of the sampling of the input which is more effective for higher rates. As discussed in the theoretical derivation, the actual effective sampling rate can be increased by adding spike trains of several motor units, to obtain the CST signal. According to the theory, the values of correlation from simulated and experimental spike trains were generally higher when the CST was used instead of individual motor unit pairs and when low-pass filtering was applied (low-pass filtering decreases the bandwidth and thus the signal is more effectively sampled). Figure 4 shows the averaged normalized cross-correlation values (500 pairs randomly selected) for different numbers of spike trains and filters, both reported for simulation and experimental results (for subject #7 with the greatest number of detected motor units, all pairs). The *x*-axis reports the number of pooled spike trains used for the calculation of the reported indexes of correlation. For the simulations, when more than 4–5 spike trains were used, the rate of change of the correlation was decreasing considerably, showing approximately a saturation value in the frequency range 0–5 Hz (coherence, Hann windows of 150 and 400 ms). Additional motor neuron spike trains increased the correlation measures by few percents only. Moreover, the variability of the estimates, as assessed by the standard deviation of the measures, decreased with increasing number of motor unit spike trains used in the calculation. This is the result of increasing accuracy in the sampling and better averaging of the independent components of the synaptic inputs. The experimental results confirmed the simulations, with generally higher levels of cross-correlation estimated when increasing the number of spike trains. Only the results for subject #7 are shown because it was the one with the greatest number of motor units identified. The results from the other subjects confirmed the trends although the number of detected units in those cases was not sufficient to reach the saturation level.

**Figure 5:**
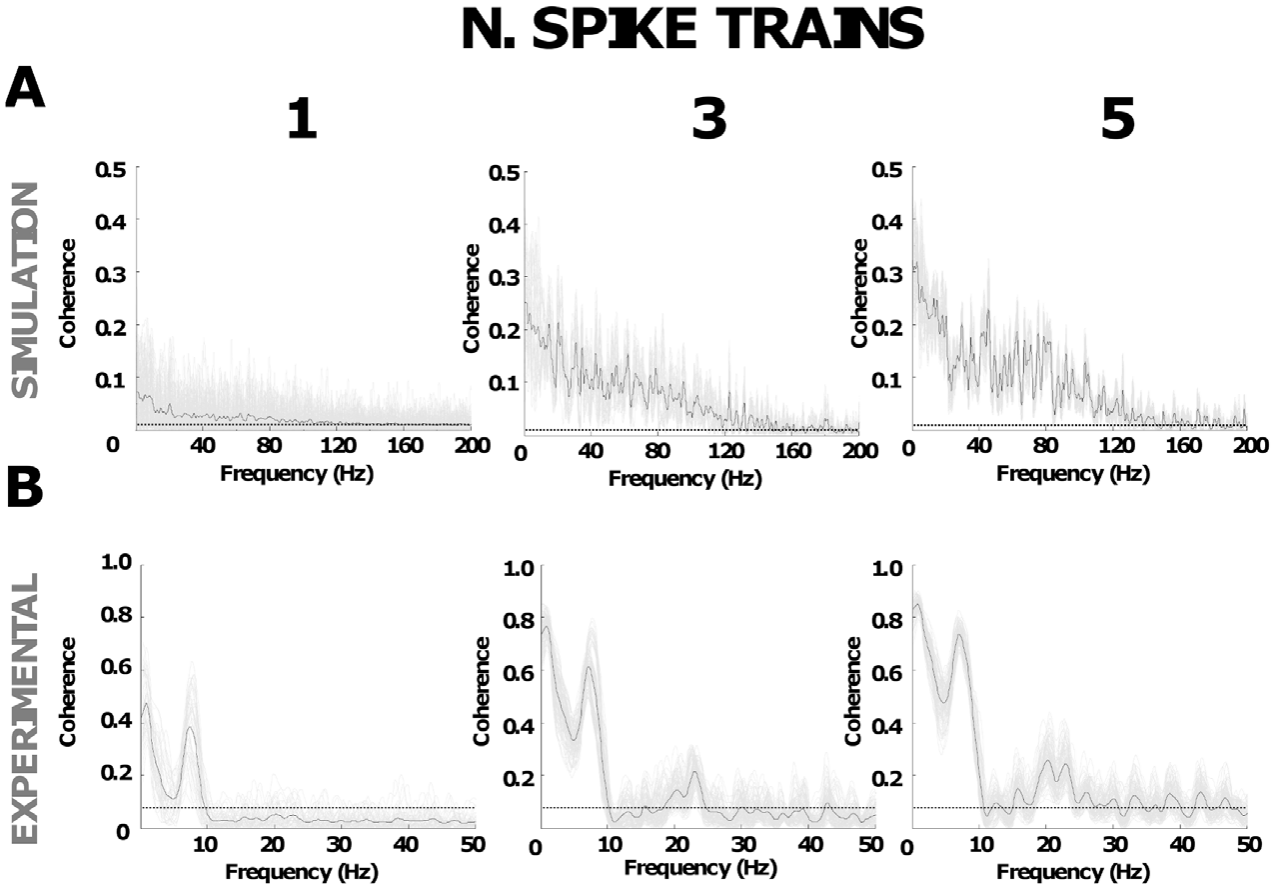
Coherence functions using pooled spike trains. A, magnitude of coherence for the simulation data (band-pass common synaptic Gaussian noise in the range 0–100 Hz) using 1, 3 and 5 pooled spike trains. Single pair combinations (light grey) and average using all available combinations (black line) B, same results for the experimental motor unit spike trains recorded from subject n. 2. The confidence level is shown with a dashed line.

The effect of the increased sampling in the pooling of several spike trains is evident when the coherence measure is applied to investigate the frequency content of the common inputs. Figure 5 shows that an increased number of spike trains changes the estimation of the coherence between CSTs and increases the bandwidth of the coherence function. Fig. 5A shows the estimation of the coherence between CST for the simulated signals. In these examples, when referring to one spike train, we mean the analysis of coherence between two CSTs each made of one spike train. The same interpretation is valid for more than one spike train. Using only one spike train in the CST, the estimated coherence calculated for 100 pairs (grey lines) and averaged (black line) showed a lower magnitude and smaller bandwidth compared with the case where 3 or 5 spike trains were used. Fig. 5B shows similar results for the experimental data recorded from subject #2. The coherence function calculated using one spike train in the CST shows common components at very low frequencies (<2−3 Hz) that are related to the low-frequency components of the control (common drive) and a second clear peak at 7.3 Hz, that corresponds approximately to the oscillation peak in the force signal (7.5 Hz in this example). No other peaks were visible over the confidence level. However, when 3 and 5 spike trains were used in the CST, significant coherence in the beta frequency band (15–30 Hz), a frequency band commonly found in EEG-EMG coherence analysis (Halliday et al. 1998), was clearly visible with an overall increase in the magnitude of the coherence for all frequencies. Moreover, the variability of the estimate was greater when only a pair of spike trains was used, as shown by the grey lines. The analysis performed across all subjects showed a similar trend. A significant peak at 5–10 Hz was indeed evident in all recordings and for the three combinations of spike trains (mean frequency, 7.5±1.2 Hz). Over all subjects, the magnitude of this peak increased by 117±40% when 3 motor unit spike trains were used compared with only one, and by 163±38% for 5 spike trains. Moreover, significant coherence in the beta band was also evident only when more than one motor unit spike train was used. Indeed the mean magnitude of the coherence in the frequency band 15–30 Hz was 1.6±0.2 times greater using 5 motor unit spike trains compared to one motor unit spike train. These results are the frequency-domain counterparts of the trends observed for the time-domain analysis.

### Effect of Filtering

As described in the theoretical part, the transfer function of the filter applied to the pairs of spike trains for the quantification of common inputs influences the estimated correlation (time domain).

**Figure 6:**
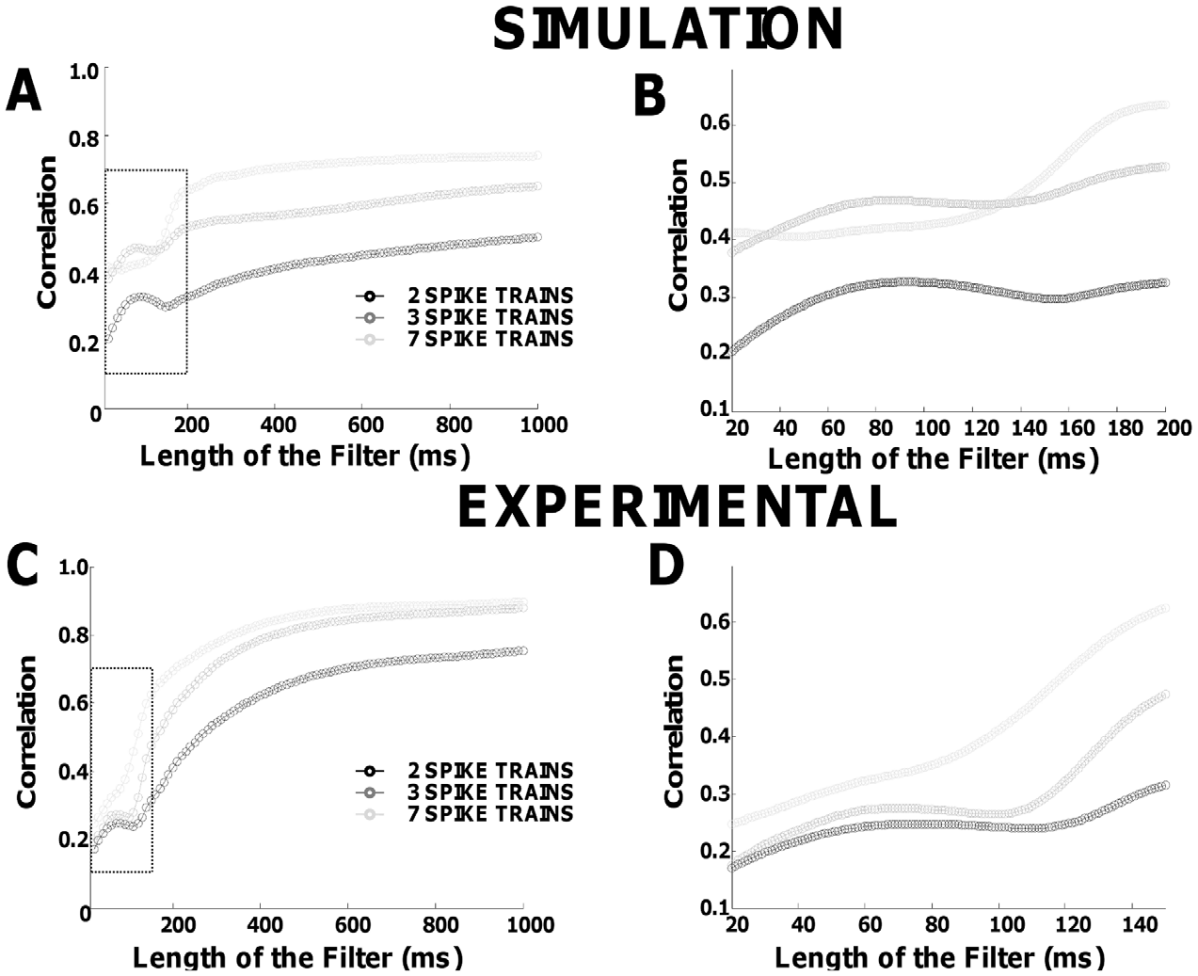
Peak of correlation magnitude between spike trains filtered with Hann windows of different lengths. A, simulations results using combinations of 2, 3 and 7 pooled spike trains. B, inset for the filter lengths in the range 20–200 ms. C, experimental results for subject n. 3. D, inset for the filter length in the range 20–140 ms. Values are reported as the mean across all combinations of the specified number of spike trains. Notice the non-monotonic behavior when the filter length is approximately equal to the mean inter-spike intervals (97±17 ms for simulations and 72±19 ms for experimental).

**Figure 7:**
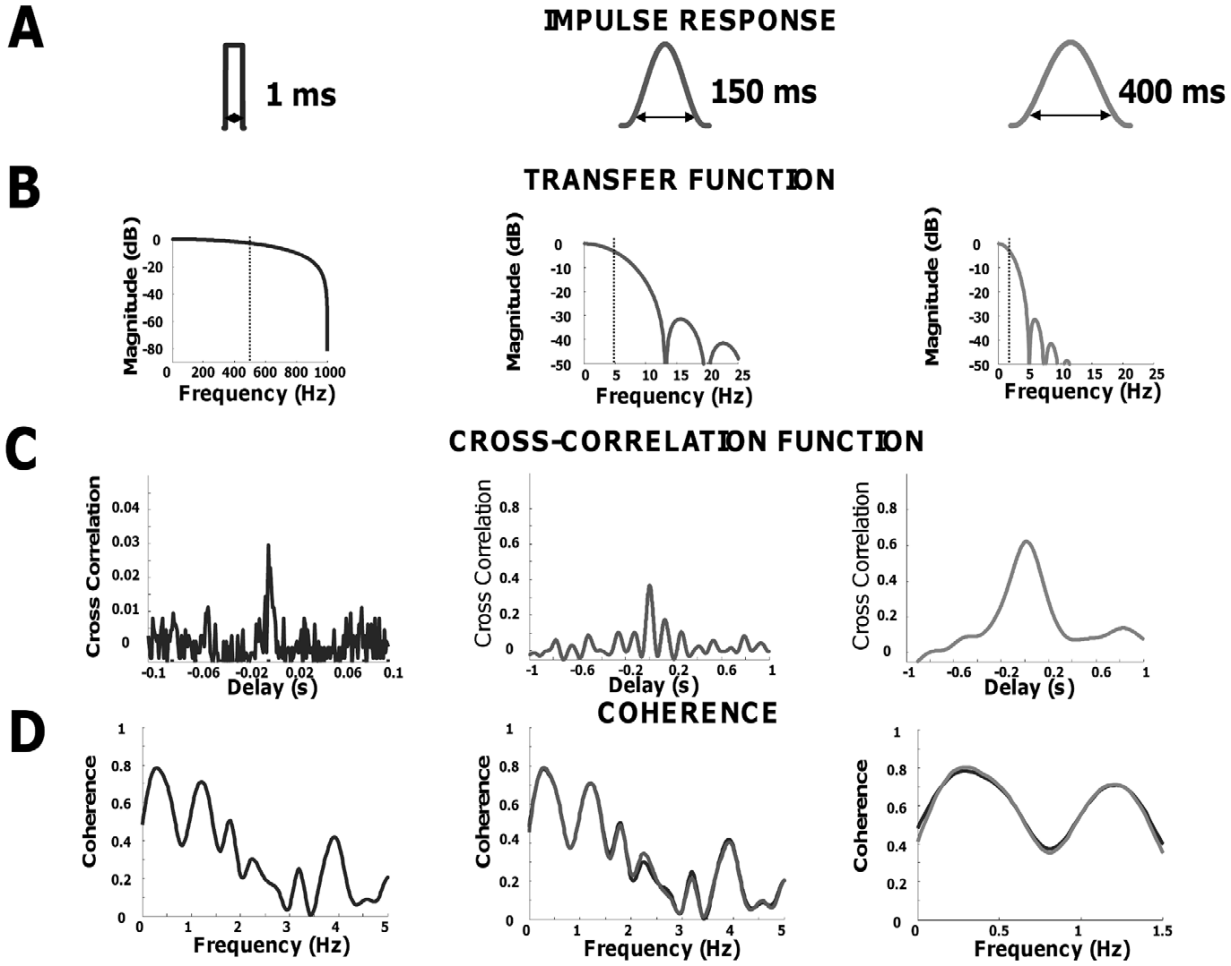
Comparison between time and frequency domain correlation for different filter lengths. A, impulse response of the three filters used in the study: rectangular window of 1 ms (equivalent to raw spike train with 1000 Hz sample rate), Hann window of 150 ms and 400 ms durations. B, transfer function for the same filters. C, cross-correlation functions calculated for two spike trains recorded from subject 7 and filtered with the above windows. D, comparison of the coherence functions for the pair of spike trains filtered with the rectangular window (black line) and the other two filters.

We systematically analyzed in simulation and experimentally, the effect of the filter length on the peak of the cross-correlation function. Figure 6 shows that the level of correlation estimated by filtered spike trains increases when the filter length increases (reduced bandwidth), although not monotonically. A similar trend could be observed for the simulated (Fig. 6A) and experimental conditions (subject #7 is represented) (Fig. 6C). The correlation values are reported for the combinations of 2 (black), 3 (dark grey), and 7 (light grey) spike trains (averaged over 100 pairs). The increase in estimated correlation with a decrease in bandwidth reflects the more efficient sampling for smaller bandwidths. When the filters had a length comparable with the inverse of the average discharge rates of the spike trains used in the calculation, however, the level of correlation reached a local maximum (Fig. 7B–D). The effect was more evident using pairs of spike trains, and it was due to the amplification of the common frequency component corresponding to the average discharge rate. Using CSTs, the effect of the discharge rate was mitigated by the increased sampling efficiency, but the transition was still evident. The overall effect on the experimental signals was perfectly predicted by the simulations.

These results indicate that the applied filter has a large impact on the time domain correlation indexes (Fig. 6). It is particularly relevant to note that the local maximum in correlation due to the average value of discharge rate imposes a large sensitivity of correlation estimates to small changes in filter length for filters with length smaller than 200 ms (corresponding to the range of physiological discharge rates).

Conversely, the coherence calculated in the frequency band of the filter used does not change with the filter. Figure 7 confirms this theoretical prediction on experimental data. The figure shows the analysis of correlation in time and frequency domain for one pair of motor unit spike trains recorded from subject #3. From the two spike trains, three cross-correlation functions were calculated with three filters (shown in Fig. 7A,B). The first filter is that usually used for the analysis of short-term synchronization [9], [10], [36] while the third is that used for the common drive analysis [7], and the second has intermediate length between the other two. Figure 7C shows the cross-correlation functions estimated after applying the three filters. Figure 7D shows the corresponding coherence functions. According to the theoretical derivation, the cross-correlation analysis in the time domain depended on the filter used, as shown by the magnitude and the shape of the three cross-correlation functions. However, the coherence function was identical in the three conditions within the bandwidth of the applied filter. The grouped data analysis confirmed this observation. The area of the coherence calculated in the frequency band described by the third filter (<1.8 Hz, the smallest frequency band) was indeed similar for the three filters (0.438±0.166 for the first, 0.442±0.168 for the second, and 0.449±0.171 for the third; P>0.05). These results indicate that different indexes of synchronization obtained from filtering the spike trains are equivalent in the frequency domain and only correspond to the analysis of different frequency bandwidths of the output spike trains. A coherence analysis of the raw spike trains therefore provides the full information on correlation strength (complete frequency range). For example, the information carried by the CDI can be extracted from the low-frequency band of the coherence estimated from the raw spike trains. Similarly, the raw cross-histogram without filtering provides the full information in the time domain.

## Discussion

We described the relation between the amount of correlation received by the motoneuron pool and the corresponding level of indexes usually applied for estimating spike train synchronization, coherence and common drive. These measures of correlation between spike trains have been often considered independent since they usually show different behaviors. However, they can all be interpreted as the subband analysis of the coherence function between spike trains. As such, they are influenced by the sampling rate and are different between each other since they address different bands in the coherence frequency axis.

Correlation analysis of motor unit spike trains aims at estimating the correlation between inputs to motoneurons from the analysis of the output spike trains. In general, the dependence of the output correlation on the characteristics of the input current to the motoneuron pool produces a bias in the estimation of the true level of input correlation [16], [20]. The problem is intuitive if it is addressed in terms of sampling process. A single motoneuron cannot reliably sample an input with frequency greater than its average discharge rate [37], [38], [55], which is very low. Therefore the level of correlation that can be estimated from the output spike trains of two motoneurons depends on the frequency content of the input current and the sampling/discharge rate, not only on the correlation in input. This problem has been addressed by normalizing the correlation indexes [9], [10], [36] in order to minimize the effect of discharge rate. Nonetheless, these normalized measures have demonstrated both a positive [32], [36], [39], [51], negative [33], or no association [36], [39], [51], [56] with the discharge rates of the analyzed spike trains, generating difficulties in the interpretation of the results. These observations are in agreement with the theoretical derivation (Eq. 1) that describes a scaling factor associating the output and input correlation that depends non-linearly on the discharge rate (note that the input current in the scaling factor is also associated to the discharge rate), so that the effect of discharge rate cannot be removed by normalization. Occasionally, normalization may reduce the dependency on discharge rate, as also observed in this study (e.g., SIP index, Fig. 3), but this is not a general observation (Table 2) and depends on the range of observed discharge rates. For this reason, it is not possible to infer dependency of correlation inputs on variables that change the discharge rate, such as force [39], since it is not possible to distinguish between the variations of input correlation and statistics of the total synaptic currents in such conditions. In this study, for example, the simulated and experimental results showed a clear relation between output correlation and discharge rates with the same synaptic current statistics and force level. Therefore, the concurrent change of both input correlation and statistics of synaptic current would result in variations of the estimated output correlation, limiting the possibility of inferring clear conclusions about the strength of the common synaptic input. These considerations are limited to the estimated strength of the correlation. Other measures extracted from the cross-correlograms between spike trains, as for example the time courses of the correlation peaks [57], may be less influenced by sampling. For estimating the strength of correlation higher motoneuron rates would be needed, but the intrinsic low discharge rates experimentally found in alpha motor neurons [58] limits this possibility. Moreover, for the same reason, the estimate of the strength of the correlation is inevitably affected by high variability.

Under the assumption of a common synaptic noise uniformly spread across the motoneuron pool [37], [41], the pooling of the spike trains extracted from several motoneurons could help to increase the sampling rate [37], [41]. However, the estimated correlation increases with the number of spike trains used for the analysis (Fig. 4). This observation is due to two effects. First, the better sampling implies a more reliable transmission of the synaptic inputs, which is the desired effect. Second, the summation of multiple spike trains determines the averaging of the output so that the independent components are relatively attenuated with respect to the common ones, which is an undesired effect that biases the result. In this way, the measure saturates (Fig. 4) [17], [18]. This limits the applicability of the technique when various conditions have to be compared since the differences will tend to vanish.

Another contribution of this study is the demonstration that different ways of assessing correlation between motor unit spike trains, such as common drive and synchronization, are actually strongly related and only differ for the bandwidth of the analysis. Time-domain correlation measures are strongly influenced by the filter used for processing the spike trains [22] which generates uncorrelated indexes when using different filters [12]. For example, a length approximately equal to the inverse of the average discharge rates of the investigated motor unit generates a large variability in the estimation (Figure 6). On the contrary, coherence measures are not influenced by the filter applied (in the filter bandwidth) (Figure 7) and thus all the information carried by different correlation indexes can simply be retrieved by analyzing different bandwidths of the coherence function. For example, the cross-correlation calculated using a short time bin, as it is typically computed for estimating the strength of short-term synchronization, describes the amount of correlation in the entire frequency range. For this reason, this measure is associated with the level of coherence present in large subbands (e.g., 15–30 Hz) [34], [51], [59], [60] that constitute a relative large part of the full frequency bandwidth. Conversely, the common drive index [7] carries information on a very small frequency band (<5 Hz) [59], [61], so that it is not surprising that it is poorly associated to the strength of synchronization [12] that represents the full frequency range. According to these results, coherence analysis in different frequency bands or time-domain cross-correlation without filtering provides the full information. Other indexes of correlation can be extracted both in time and frequency domain by filtering the cross-histograms or coherence functions, respectively. However, the coherence function (and raw cross-histogram) still depends on the number of spike trains used for the analysis so that a sufficient number of spike trains is needed for identifying all the significant peaks in the coherence function (Fig. 5) (or corresponding features in the time domain). For the same reasons as discussed for time-domain indexes, the actual peak values in the coherence function cannot be compared directly when extracted from motoneurons with different discharge rates and relative comparisons are the only possible. Interestingly, the coherence function approximates the power spectrum of the common synaptic input to motoneurons when a large number of motoneurons is used for the estimate, despite the non-linearity of each motoneuron, for the same reason that common cortical oscillations to the motoneuron pool can be extracted by coherence analysis between EEG and motoneuron spike trains [37]. We previously provided an analytical demonstration of this effect in the context of EEG-EMG coherence [37] that could be applied almost without changes also in the case of coherence between spike trains.

In conclusion, this study clarifies controversial issues in the estimation of common inputs to alpha motoneurons. It shows that an intrinsic dependency on discharge rate of these measures is expected theoretically and cannot be removed by normalization, thus the interpretation of correlation measures should be cautious when the discharge rate differs, even if some normalization is applied. The study further proves that indexes used for assessing the common input signal to motoneurons can all be extracted by the coherence analysis (or cross-histogram in time domain) of the raw spike trains. Therefore, the extraction of coherence or cross-histogram between spike trains should be preferred over the same measures obtained with pre-filtering. Furthermore, the use of populations of motor units rather than pairs improves the detection of significant frequency peaks in the coherence function. This is due to the influence of discharge rate on the absolute magnitude of coherence peaks, which also indicates that only relative comparisons of peak values are possible. These conclusions lead to the indication that common inputs to motoneurons should be investigated from the calculation of the coherence or cross-histogram functions between the unfiltered CST of populations of motoneurons. The information extracted in this way is not related to an absolute strength of the common input but to the identification of spectral frequencies or temporal correlations present in such input.
